# lncRNA‐NKILA/NF‐*κ*B feedback loop modulates laryngeal cancer cell proliferation, invasion, and radioresistance

**DOI:** 10.1002/cam4.1405

**Published:** 2018-03-23

**Authors:** Tao Yang, Shisheng Li, Jiajia Liu, Danhui Yin, Xinming Yang, Qinglai Tang

**Affiliations:** ^1^ Department of Otolaryngology Head and Neck Surgery The Second Xiangya Hospital Central South University Changsha Hunan 410011 China

**Keywords:** Feedback loop, laryngeal cancer, NF‐*κ*B, NF‐*κ*B interacting lncRNA, radioresistance

## Abstract

Laryngeal cancer is one of the most common head and neck malignant tumors and is commonly resistant to X‐ray‐based radiotherapy. NF‐*κ*B interacting lncRNA (NKILA) has been reported to serve as a tumor suppressor in several cancers through combining with NF‐*κ*B: I*κ*B complex thereby inhibiting NF‐*κ*B activation. Herein, we demonstrated a low NKILA expression in laryngeal cancer and its correlation with shorter overall survival in patients with laryngeal cancer. NKILA serves as a tumor suppressor in laryngeal cancer by suppressing laryngeal cancer cell viability and migration, whereas promoting cell apoptosis; NKILA knockdown reverses the cytotoxicity of X‐ray radiation on laryngeal cancer cells through combining with NF‐*κ*B: I*κ*B complex to inhibit I*κ*B phosphorylation, inhibit p65 nuclear translocation, and finally inhibit NF‐*κ*B activation. NF‐*κ*B binds to the promoter region of NKILA to activate its transcriptional activity, upregulated NKILA then inhibits I*κ*B phosphorylation and NF‐*κ*B activation, thus forming a negative feedback loop to sensitize laryngeal cancer cell to X‐ray radiation. In conclusion, NKILA can serve as a promising agent of enhancing the cytotoxicity of X‐ray radiation on laryngeal cancer and addressing the radioresistance of laryngeal cancer.

## Introduction

Laryngeal cancer is one of the most common head and neck malignant tumors. X‐ray radiation‐based therapy has been widely used to treat laryngeal cancer; however, due to the acquisition of radioresistance, the improvement of the survival time of patient with laryngeal cancer and preservation of laryngeal function is limited 1, 2, 3, 4, 5, 6. Though several factors, such as p53 alternation, dysregulation of miRNAs, overexpression of Bcl‐2, NF‐*κ*B, or COX2 4, 7, 8, 9, 10, are reportedly related to the radioresistance of laryngeal cancer, the detailed mechanism by which the radioresistance of laryngeal cancer is induced still remains unclear.

Recently, a large amount of researches revealed the potential value of another mechanism of long noncoding RNA (lncRNA)‐mediated nonmutational gene function regulation 11, 12. lncRNA expression dysregulation has been reported in many kinds of cancers; they can serve as tumor suppressors or oncogenes or both, depending on circumstance 13, 14. NF‐*κ*B interacting lncRNA (NKILA) has been reported to be up regulated by NF‐*κ*B in many cancers, including breast cancer 15, malignant melanoma 16, and nonsmall cell lung cancer 17. Liu et al. 15 demonstrated that NKILA can combine with NF‐*κ*B: I*κ*B complex, thus suppressing the cell migration and invasion of breast cancer, inhibiting IKK‐induced I*κ*B phosphorylation and finally blocking the activation of NF‐*κ*B signaling. Similarly, NKILA plays a crucial role in suppressing tongue squamous cell carcinoma cell migration and invasion through hindering I*κ*B phosphorylation, NF‐*κ*B activation, and the following EMT process 18. However, the role and molecular mechanism of NKILA in laryngeal cancer progression still remain unclear.

More importantly, NF‐*κ*B itself plays a key role in laryngeal cancer. In laryngeal cancer cells, NF‐*κ*B can induce excessive centrosome amplification, which can drive chromosomal instability, a major source of tumor initiation 19. Alterations in the cellular microenvironment that propel metastatic events in laryngeal cancer include induction of downstream signaling mediated by NF‐*κ*B and Src tyrosine kinase 20. Moreover, several anticancer means in laryngeal cancer are mediated by NF‐*κ*B through inhibiting NF‐*κ*B activity 21, 22. Based on the previous findings, we hypothesized that NKILA might play a potential role in laryngeal cancer in a NF‐*κ*B‐dependent manner.

Furthermore, the functions of lncRNAs in determining drug sensitivity/resistance have been more and more reported 11, 23, 24. Herein, we evaluated the expression NKILA in laryngeal cancer tissues, assessed the functional role of NKILA in tumor cell proliferation, migration, apoptosis, and radioresistance to X‐ray radiation; moreover, we validated NKILA regulation of NF‐*κ*B signaling and the combination of NKILA and NF‐*κ*B: I*κ*B complex, and further investigated whether NKILA affected laryngeal cancer radioresistance through NF‐*κ*B signaling; finally, we investigated the mechanism by which NF‐*κ*B regulated NKILA expression in tumor cells. Taken together, we provided experimental and theoretical basis for understanding the mechanism of NKILA‐mediated laryngeal cancer radioresistance regulation.

## Material and Method

### Tissues, cell line, and cell culture

With the approval of the Ethic Committee of The Second Xiangya Hospital, Central South University, we collected 65 paired Laryngeal cancer tissues as well as the adjacent normal tissues. All samples were obtained from patients who underwent surgical resection at The Second Xiangya Hospital, Central South University (Changsha, China). All the tissue samples were snap‐frozen and stored at −80°C in liquid nitrogen. The clinical features of patients are listed in Table 1. Univariate and multivariate analyses of factors related to overall survival using the Cox proportional hazard model are listed in Table 2.

**Table 1:** Correlation between NKILA expression and clinicopathological features

Parameters	Group	NKILA expression		*P*‐value
		High	Low	
TNM	I + II	21	8	0.002
	III + IV	12	24	
Lymph node metastasis	N0	16	5	0.005
	N1–N4	17	27	
Age (years)	>60	19	13	0.172
	≤60	14	19	
Sex	Female	10	6	0.28
	Male	23	26	
Location	Glottic	17	17	0.897
	Supraglottic	16	15	
Differentiation	Moderate or poor	14	15	0.718
	Well	19	17	

**Table 2:** Univariate and multivariate analysis for factors related to overall survival using the Cox proportional hazard model

Characteristics	Univariate analysis			Multivariate analysis		
	*P*‐value	HR	CI 95%	*P*‐value	HR	CI 95%
NKILA						
High vs. low	0.010	0.309	0.127–0.754	0.048	0.389	0.152–0.993
TNM						
I + II vs. III + IV	0.083	0.455	0.187–1.108	0.575	0.759	0.289–1.993
Lymph node metastasis						
N0 vs. N1–N4	0.071	0.370	0.126–1.089	0.381	0.591	0.182–1.917
Age (years)						
≥50 vs. <50	0.348	1.486	0.650–3.393	N.A		
Sex						
Female vs. male	0.372	0.612	0.208–1.799	N.A		
Location						
Glottic vs. Supraglottic	0.574	1.267	0.555–2.891	N.A		
Differentiation						
Moderate or poor vs. well	0.564	1.273	0.561–2.887	N.A		

HEp‐2 and TU212 cells were purchased from the American Type Culture Collection (ATCC, Manassas, VA) and Xiangya Central Experiment Laboratory (Hunan, China), respectively, and cultured in RPMI‐1640 (Gibco‐BRL, Gaithersburg, MD) supplemented with 10% heat‐inactivated fetal bovine serum (Hyclone, Logan, UT), 100 U/mL penicillin, and 100 U/mL streptomycin at 37°C in a 5% CO_2_ atmosphere. Cells were trypsinized and harvested after reaching 80–90% confluence.

### Cell treatment

For X‐ray radiation, HEp‐2 and TU212 cells were exposed to different doses of X‐ray radiation (2, 4, 6, 8, and 10 Gy) for different times (24, 48, and 72 h). X‐ray radiation was performed on accelerator linear (Clinac 23EX; Varian Company, Palo Alto, CA). Source‐skin distance was 100 cm, radiation field was 35 × 35 cm^2^, a single energy was 6 MV X‐ray, and dose rate was 500 MU/min.

To inhibit NF‐*κ*B nuclear translocation, 5 *μ*mol/L JSH (Millipore, MA) was added to culture media 30 min prior to the lentivirus infection. Cells were harvested for further experiments.

For TNF‐*α* treatment, cells were treated with 10 ng/mL TNF‐*α* (Sigma, St. Louis, MI) for 30 min to activate NF‐*κ*B. Cells were harvested for further experiments.

### Lentivirus production, titration, and infection

To generate NKILA‐inhibiting or overexpressing or control lentivirus, the plasmids encoding shRNA NKILA‐1 or shRNA NKILA‐2 or shRNA NKILA‐3 or LV‐NKILA or the control scrambled sequence were cotransfected into 293FT cells (ATCC) together with the plasmids pHelper1.0 and pHelper2.0 (Genechem, Shanghai, China) that contain the elements required for virus packaging, using Lipofectamine 2000 (Invitrogen, Waltham, MA) according to the manufacturer's instructions. The culture supernatants containing lentivirus were harvested and concentrated by ultracentrifugation, and the viral titers were determined. To perform lentiviral infection, the target cells were plated at 40–50% confluence and incubated overnight (16 h). On the day of infection, the culture medium was replaced with viral supernatant at an appropriate titer (1.5 mL/well), incubated at 37°C for 10 h, and then the viral supernatant was replaced with fresh media. Forty‐eight hours later, the infected cells were selected using puromycin (2 mg/mL). After 5 days of selection, NKILA inhibition and overexpression efficiency were determined by real‐time PCR assays.

### Cell transfection

P65 knockdown or overexpression was achieved by transfection of si‐p65 or pCMV‐p65 vector (Genepharm, Pallini, Greece) using Lipo2000 (Invitrogen) according to the manufacturer's instructions.

### RNA extraction and SYBR green quantitative PCR analysis

We extracted total RNA from cells using Trizol reagent (Invitrogen), and NKILA expression was measured by the SYBR green qPCR assay (Takara, Dalian, China). The data were processed using the 2^−ΔΔCT^ method.

### MTT assay

A modified MTT assay was used to evaluate cell viability. Twenty‐four hour after seeding into 96‐well plates (5000 cells per well), cells were infected with different lentivirus. Forty‐eight hour after infection, 20 *μ*L MTT (at a concentration of 5 mg/mL; Sigma‐Aldrich, St. Louis, MI) was added, and the cells were incubated for an additional 4 h in a humidified incubator; 200 *μ*L DMSO was added after the supernatant discarded to dissolve the formazan. OD_490 nm_ value was measured. The viability of the nontreated cells (control) was defined as 100%, and the viability of cells from all other groups was calculated separately from that of the control group.

### BrdU incorporation assay

DNA synthesis in proliferating cells was determined by measuring 5‐bromo‐2‐deoxyuridine (BrdU) incorporation. BrdU assays were performed at 24 h and 48 h after infection. After seeding the infected cells in 96‐well culture plates at a density of 2 × 10^3^ cells/well, they were cultured for 24 h or 48 h, and incubated with a final concentration of 10 *μ*mol/L BrdU (BD Pharmingen, San Diego, CA) for 2 h to 24 h. When the incubation period ended, we removed the medium, fixed the cells for 30 min at RT, and incubated them with peroxidase‐coupled anti‐BrdU antibody (Sigma‐Aldrich) for 60 min at RT, washed them three times with PBS, incubated the cells with peroxidase substrate (tetramethylbenzidine) for 30 min, and measured the absorbance values at 450 nm. Background BrdU immunofluorescence was determined in cells not exposed to BrdU but stained with the BrdU antibody.

### In vitro migration assays

Cells (5 × 10^5^) were planted on the top side of polycarbonate Transwell filters (without Matrigel for Transwell assay) (Cell Biolabs, Inc., Santiago, MN). For Transwell migration assays, cells were suspended in medium without serum and medium without serum was used in the bottom chamber. The cells were incubated at 37°C for 48 h. The nonmigratory cells in the top chambers were removed with cotton swabs. The migrated cells on the lower membrane surface were fixed in 100% methanol for 10 min, air‐dried, then stained with DAPI (Beyotime Institute of Biotechnology, Haimen, China), and counted under a microscope.

### Flow cytometer assay

For apoptosis analysis, quantification of apoptotic cells was performed with Annexin V‐FITC apoptosis detection kit (Keygen, China). Briefly, the cell samples were harvested with 0.25% trypsin without EDTA after 48 h of infection and then washed twice with ice‐cold PBS and resuspended in 500 *μ*L binding buffer. Then cells were incubated with 5 *μ*L Annexin V‐FITC‐specific antibodies and 5 *μ*L propidium iodide (PI) then incubated for 15–20 min in dark and detected by BD Accuri C6 flow cytometer (BD) with the excitation wavelength of Ex = 488 nm and emission wavelength of Em = 530 nm. Each experiment was repeated three times in triplicate.

### Western blot analysis

The protein levels of p65, IKK, p‐IKK*β*, I*κ*B*α*, and p‐I*κ*B*α* in laryngeal cancer cells were detected by performing immunoblotting. We lysed cultured or transfected cells in RIPA buffer with 1% PMSF and loaded protein onto an SDS‐PAGE minigel and transferred them onto PVDF membrane. The blots were probed with the following antibodies: anti‐p65 (ab16502; Abcam, Cambridge, MA), anti‐IKK (ab178870; Abcam), anti‐p‐IKK*β* (phospho Y199; ab59195; Abcam), anti‐I*κ*B*α* (#9242; Cell Signaling Technology, Danvers, MA), anti‐p‐I*κ*B*α* (Ser 32; sc‐7977; Santa Cruz, Dallas, TX), anti‐*β*‐actin (ab6276; Abcam), and anti‐Lamin B (ab133741; Abcam) at 4°C overnight, subsequently incubated with HRP‐conjugated secondary antibody (1:5000). ECL substrates were used to visualize signals (Millipore, MA). *β*‐actin was used as an endogenous protein for normalization.

### RNA Immunoprecipitation (RIP)

RNA immunoprecipitation was performed using Magna RIP RNA‐Binding Protein Immunoprecipitation Kit (17‐700; Millipore, MA) according to manufacturer's instructions. RNA for in vitro experiments was transcribed using T7 High YieldRNA Synthesis Kit (E2040S, NEB) according to manufacturer's instructions. IgG, p65, and I*κ*B*α* RNA levels in the immunoprecipitates were measured by qRT‐PCR.

### Chromatin immunoprecipitation (ChIP)

The treated cells were cross‐linked with 1% formaldehyde, sheared to an average size of 400 bp DNA, and immunoprecipitated using antibodies against p65 (anti‐p65, ab16502; Abcam). A positive control antibody (RNA polymerase II) and a negative control nonimmune IgG were used to demonstrate the efficacy of the kit reagents (Epigentek Group Inc., Farmingdale, NY, P‐2025‐48). The immunoprecipitated DNA was subsequently cleaned, released, and eluted. The eluted DNA was used for downstream applications, such as ChIP‐PCR. The fold enrichment (FE) was calculated as the ratio of the amplification efficiency of the ChIP sample to that of the nonimmune IgG. The amplification efficiency of RNA polymerase II was used as a positive control. FE% = 2 (IgG CT‐Sample CT) × 100%.

### Luciferase activity

HEK293 cells (ATCC) were cultured overnight after being seeded into a 24‐well plate. A wild‐type and mutated NKILA promoter (wt‐NKILA and mut‐NKILA containing a mutation in any or both of the two predicted sites of the p65‐responsive element, p65RE) luciferase reporter gene vector were constructed. After cultured overnight, cells were transfected with the indicated vectors in the presence or absence of TNF‐*α* (10 ng/mL for 24 h), an activator of p65, respectively. Luciferase assays were performed 48 h after transfection using the Dual Luciferase Reporter Assay System (Promega, WI).

### Immunofluorescence staining

For the detection of p65 nuclear translocation, cells (1 × 10^5^ per well) were seeded in six‐well glass‐bottomed plate. After the cells were treated, they were fixed in 4% paraformaldehyde for 30 min and then permeabilized with 0.2% Triton X‐100 for 15 min. Nonspecific binding sites were blocked with 1% BSA in PBS for 2 h. Then, the cells were treated with primary antibody specific to p65 (ab16502; Abcam, 1 *μ*g/mL) overnight at 4°C. Thereafter, the cells were incubated with TRITC‐conjugated secondary antibody (Beyotime, China) for 1 h at dark. DAPI (Beyotime, China) was used to stain nuclei before capturing images. The images were acquired using a fluorescence microscope (Nikon, Japan). The green fluorescence indicated p65 expression, and the blue fluorescence indicated nuclei.

### Statistical analyses

Data from three independent experiments were expressed as the mean ± SD and processed using SPSS17.0 statistical software (IBM Corporation, Armonk, NY). Differences between two groups were compared using the Student's paired test. Differences among more than two groups in the above assays were estimated using one‐way ANOVA. A *P*‐value <0.05 was considered significant.

## Results

### The expression of NKILA and its correlation with the prognosis of patients with laryngeal cancer

**Figure 1:**
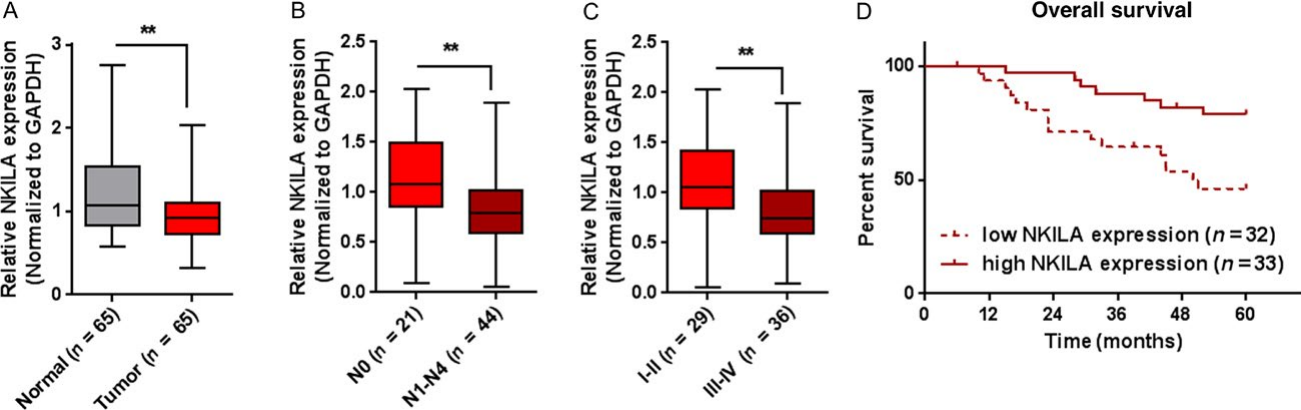
The expression of NKILA and its correlation with the prognosis of patients with laryngeal cancer (A) The expression of NKILA in 65 paired laryngeal cancer and adjacent normal tissues was determined using real‐time PCR assays. (B and C) The expression levels of NKILA in tumor tissues were analyzed according to N staging or clinic staging. (D) Kaplan–Meier overall survival curves for 65 patients with laryngeal cancer classified according to relative NKILA expression level. The data are presented as mean ± SD of three independent experiments. **P <* *0.01.

In order to evaluate the role of NKILA in laryngeal cancer, we first determined NKILA expression in normal and laryngeal cancer tissues using real‐time PCR assays. Consistent with previous studies in other cancers 16, 17, 18, NKILA expression was dramatically down regulated in laryngeal cancer tissues compared to that in normal tissues (Fig. 1A). In addition, we analyzed NKILA expression in tissues grouping by N staging or clinical staging. The results showed that NKILA expression was lower in samples of advanced N stages (N1–N4) or clinical stages (III‐IV) (Fig. 1B and C). We then divided these samples into two groups according to NKILA expression: high NKILA expression group (above the median NKILA expression, *n* = 33) and low NKILA expression group (below the median NKILA expression, *n* = 32) (Table 1). According to clinicopathological feature analysis, lower NKILA expression was observed more frequently in patients with advanced TNM stages (*P *=* *0.002) and larger tumor size (*P *=* *0.005). The overall survival and pathologic features of 65 patients were analyzed using the Cox risk proportional regression model. Univariate analysis showed that TNM stage, NKILA expression, and lymph node metastasis caused more obvious differences in overall survival time; multivariate analysis showed that low NKILA expression was of high risk (HR = 0.389, 95% CI = 0.152–0.993) (Table 2). Kaplan–Meier overall survival (OS) analysis was performed to analyze the overall survival time of patients with laryngeal cancer according to the above grouping. The results showed that the OS of the patients with lower NKILA expression was significantly shorter than that of the patients with higher NKILA expression (Fig. 1D). The data suggest that NKILA might serve as a tumor suppressor laryngeal cancer.

### The effect of NKILA on laryngeal cancer cell proliferation and migration

**Figure 2:**
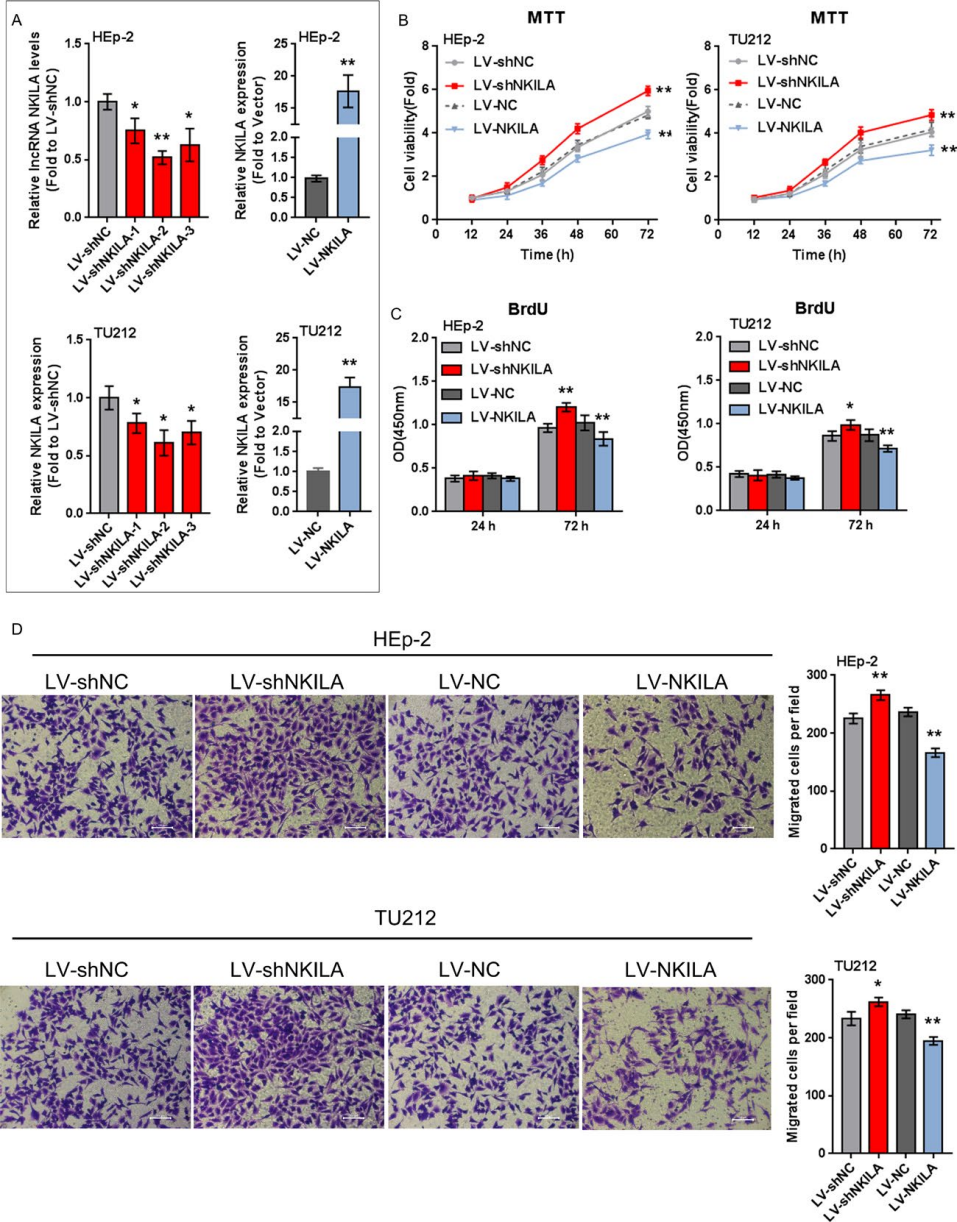
The effect of NKILA on laryngeal cancer cell proliferation and migration (A) HEp‐2 and TU212 cells were infected with LV‐shNKILA‐1 or LV‐shNKILA‐2 or LV‐shNKILA‐3 or LV‐NKILA to achieve NKILA expression, as confirmed using real‐time PCR assays. (B) The cell viability was determined using MTT assays. (C) The DNA synthesis capability was determined using BrdU assays. (D) The cell migration ability was determined using Transwell assays. The data are presented as mean ± SD of three independent experiments. *P <* *0.05, **P <* *0.01.

Two laryngeal cancer cell lines, HEp‐2 and TU212, were infected with NKILA‐inhibiting (LV‐shNKILA‐1, LV‐shNKILA‐2, or LV‐shNKILA‐3) or overexpressing lentivirus (LV‐NKILA) to achieve NKILA knockdown or overexpression, as confirmed using real‐time PCR assays. The results showed that NKILA knockdown or overexpression was successfully achieved (Fig. 2A); NKILA expression was more strongly inhibited by LV‐shNKILA‐2; thereby, LV‐shNKILA‐2 was selected as shRNA for NKILA knockdown (Fig. 2A). Next, the cell viability, DNA synthesis capability, and migration ability of HEp‐2 and TU212 cells were determined using MTT, BrdU, and Transwell assays. NKILA overexpression significantly suppressed the cell viability, DNA synthesis capability, and migration ability of laryngeal cancer cells; on the contrary, NKILA knockdown exerted an opposing role: NKILA knockdown dramatically promoted laryngeal cancer cell viability, DNA synthesis capability, and migration ability (Fig. 2B–D). The data indicate that NKILA can suppress laryngeal cancer cell proliferation and migration; however, the underlying mechanism still remains unclear.

### Determination of radiation dose

**Figure 3:**
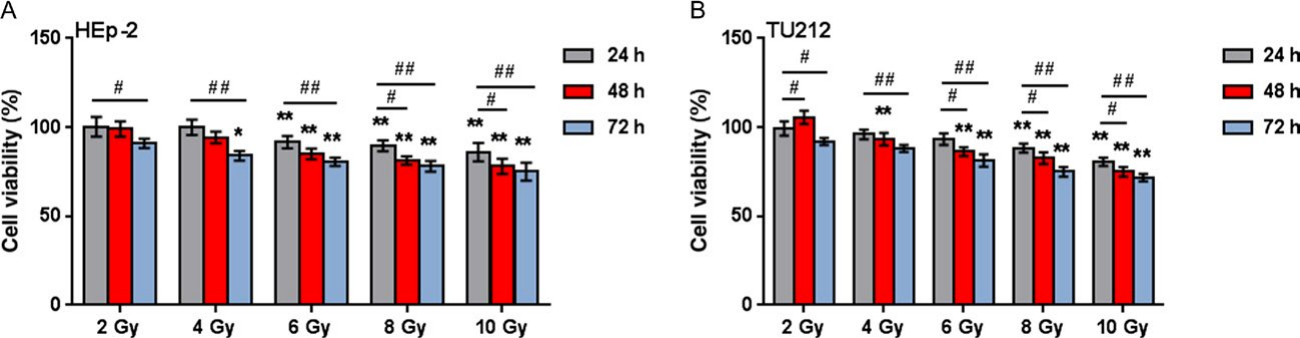
Determination of radiation dose (A and B) HEp‐2 and TU212 cells were exposed to 2, 4, 6, 8, or 10 Gy X‐ray radiation for 24, 48, or 72 h; the cell viability was determined using MTT assays. The data are presented as mean ± SD of three independent experiments. *P <* *0.05, **P <* *0.01, compared to 2 Gy treatment for the same timing group; #*P *<* *0.05, ##*P *<* *0.01, compared to same dose treatment for 24 h group.

We have revealed that NKILA suppresses the cell proliferation and migration of laryngeal cancer cells; next, we assessed the effect of NKILA on the radioresistance of laryngeal cancer cells. HEp‐2 and TU212 cells were exposed to different doses of X‐ray radiation (2, 4, 6, 8, and 10 Gy) for different times (24, 48, and 72 h); the cell viability was determined using MTT assays. The results showed that 2, 4, and 6 Gy could not cause significant differences in cell viability of both HEp‐2 and TU212 cells during all treatment times (Fig. 3A and B); on 24, 48, and 72 h after 8 Gy or 10 Gy radiation, the cell viability was significantly suppressed (#*P *<* *0.05, Fig. 3A and B); the cell viability was significantly suppressed on 72 h after any dose of X‐ray radiation (#*P *<* *0.05, ##*P *<* *0.01, Fig. 3A and B).

### The effect of NKILA on the radioresistance of laryngeal cancer cell

**Figure 4:**
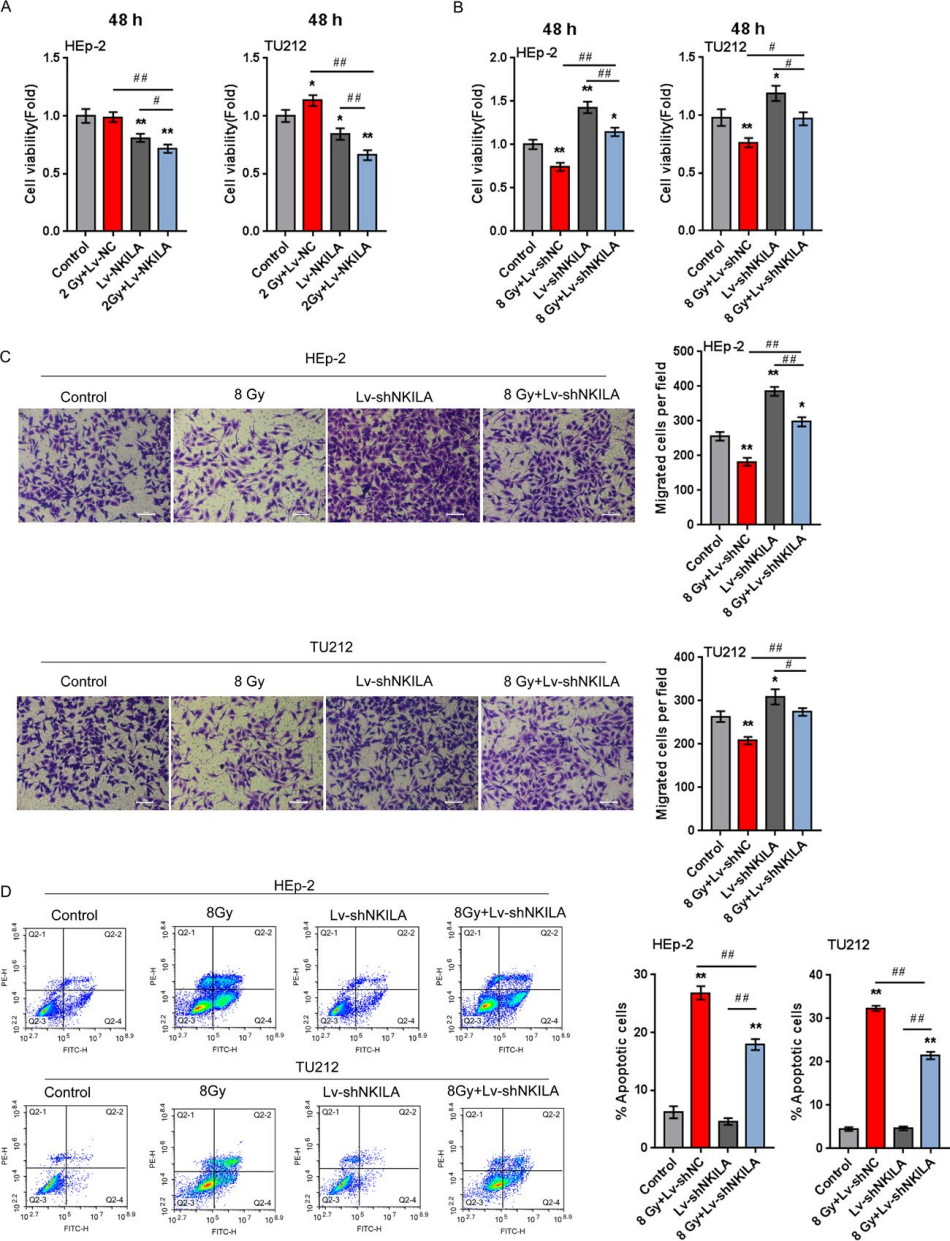
The effect of NKILA on the radioresistance of the laryngeal cancer cell (A) HEp‐2 and TU212 cells were infected with LV‐NC or LV‐NKILA in the presence or absence of 2 Gy X‐ray radiation for 48 h; the cell viability was determined using MTT assays. (B) HEp‐2 and TU212 cells were infected with LV‐shNC or LV‐shNKILA in the presence or absence of 8 Gy X‐ray radiation for 48 h; the cell viability was determined using MTT assays. (C) Cell migration was determined using Transwell assays. (D) Cell apoptosis was determined using flow cytometer assays. The data are presented as mean ± SD of three independent experiments. *P <* *0.05, **P <* *0.01, compared to control group; #*P *<* *0.05, ##*P *<* *0.01, compared to 8 Gy + LV‐shNC group.

As we have found that 2 Gy treatment caused only slight downregulation or even upregulation of cancer cell viability and that 8 Gy treatment for 48 h leads to significant inhibition of laryngeal cancer cell viability; next, we investigated the effect of NKILA on the radioresistance of laryngeal cancer. HEp‐2 and TU212 cells were infected with LV‐NKILA or LV‐shNKILA and exposed to 2 Gy or 8 Gy X‐ray radiation for 48 h; the cell viability was then detected. The results showed that the cell viability was not changed or promoted by 2 Gy irradiation whereas significantly suppressed by 8 Gy radiation; the cell viability was significantly suppressed by NKILA overexpression whereas promoted by NKILA knockdown; the cellular effect of 2 Gy or 8 Gy radiation on laryngeal cancer cell viability could be partially reversed by NKILA overexpression or NKILA knockdown, respectively (Fig. 4A and B).

Furthermore, the combined effect of NKILA knockdown and 8 Gy irradiation on cell migration and apoptosis was determined. Contrary to tumor cell viability and migration, laryngeal cancer cell apoptosis was significantly promoted by 8 Gy radiation while slightly reduced by NKILA knockdown; the promotive effect of X‐ray radiation on tumor cell apoptosis could be partially reversed by NKILA knockdown (Fig. 4C). The data indicate that NKILA knockdown can enhance the resistance of laryngeal cancer to X‐ray radiation.

### NKILA inhibits NF‐*κ*B signaling through inhibiting the phosphorylation of I*κ*B

**Figure 5:**
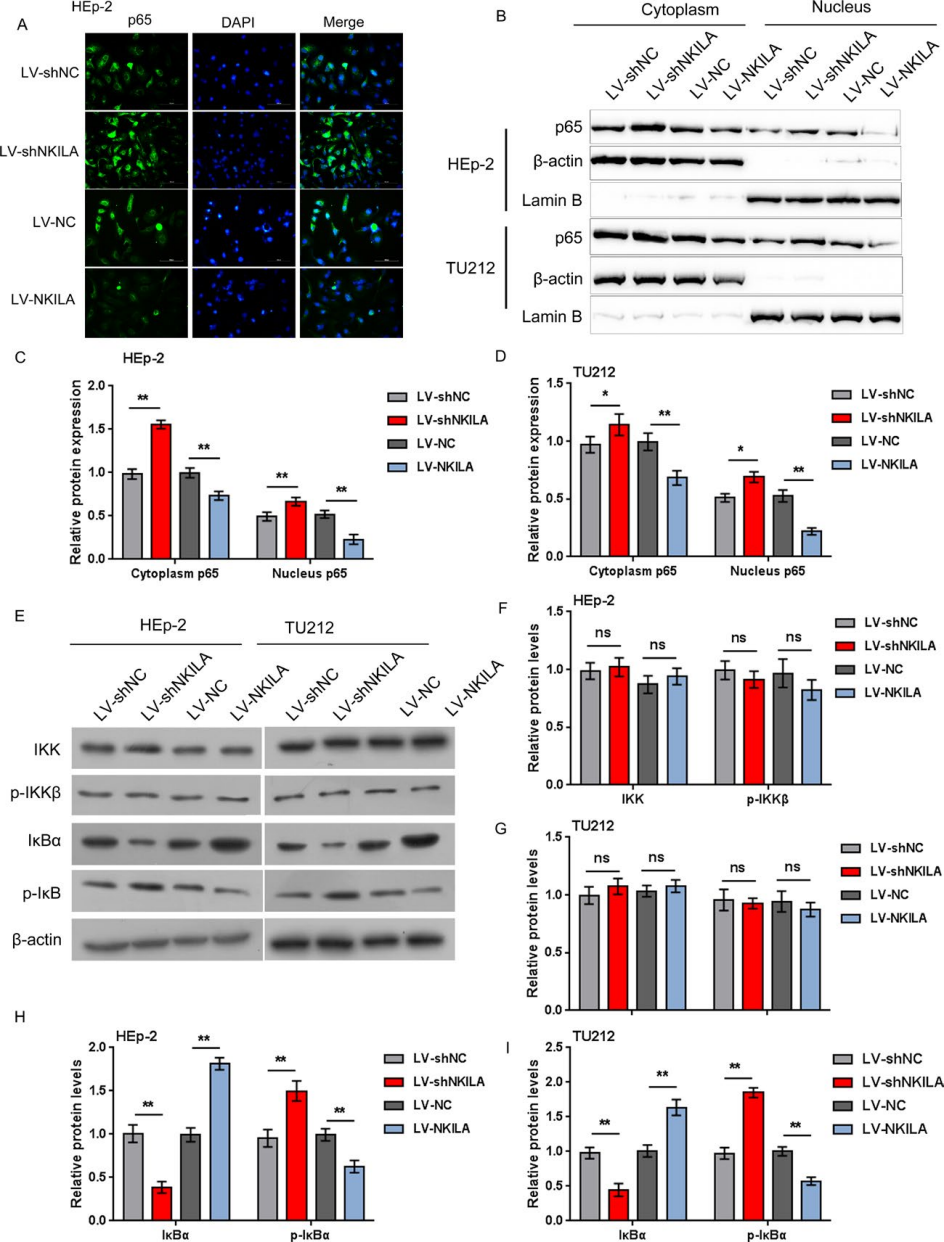
NKILA inhibits NF‐*κ*B signaling through inhibiting the phosphorylation of I*κ*B HEp‐2 and TU212 cells were infected with LV‐shNKILA or LV‐NKILA;* β*‐actin and Lamin B were used for endogenous control for cytoplasm or nucleus protein, respectively. (A) The nuclear translocation of p65 was detected using immunofluorescence assays; (B–D) the protein levels of the cytoplasm and the nucleus p65 were determined using Western blot assays; (E–I) the protein levels of IKK, p‐IKK *β*, I*κ*B*α*, and p‐I*κ*B*α* were determined using Western blot assays. The data are presented as mean ± SD of three independent experiments. *P <* *0.05, **P <* *0.01.

NKILA reportedly hinders the activation of NF‐*κ*B signaling pathway through combining with the NF‐*κ*B/I*κ*B complex via inhibiting I*κ*B, which stabilizes the complex 15. Herein, we validated whether NKILA can inhibit NF‐*κ*B signaling through the inhibition of I*κ*B in laryngeal cancer cell lines. HEp‐2 and TU212 cells were infected with LV‐shNKILA or LV‐NKILA; the location and protein levels of NF‐*κ*B signaling‐related factors were determined using immunofluorescence and Western blot assays. The results showed that NKILA knockdown promoted, whereas NKILA overexpression blocked p65 nuclear translocation (Fig. 5A). The cytoplasm and nucleus NF‐*κ*B (p65) protein levels were dramatically increased by NKILA knockdown, whereas reduced by NKILA overexpression in HEp‐2 and TU212 cells (Fig. 5B–D). Moreover, NKILA knockdown remarkably reduced I*κ*B*α* protein expression, whereas increased p‐I*κ*B*α* protein expression; NKILA overexpression increased I*κ*B*α* protein expression while reduced I*κ*B*α* protein expression; in the meantime, neither NKILA knockdown nor NKILA overexpression caused significant differences in IKK and p‐IKK*β* protein levels (Fig. 5E–I). The data indicate that NKILA overexpression can inhibit NF‐*κ*B signaling through inhibiting the phosphorylation of I*κ*B*α*.

### NKILA combines with NF‐*κ*B: I*κ*B forming stable complex

**Figure 6:**
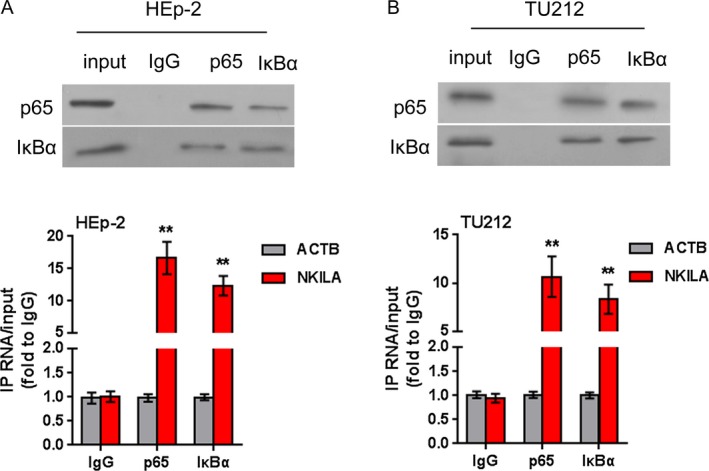
NKILA combines with NF‐*κ*B: I*κ*B to form stable complex (A‐B) Binding of NKILA to p65 and I*κ*B*α* complex in HEp‐2 and TU212 cells, shown by RNA immunoprecipitation and real‐time PCR assays. ACTB was used as negative control. The data are presented as mean ± SD of three independent experiments. **P <* *0.01.

NKILA interacting with NF‐*κ*B: I*κ*B complex, thus inhibiting the phosphorylation of I*κ*B in breast cancer cells 15; herein, we validated whether NKILA could combine with NF‐*κ*B: I*κ*B complex in laryngeal cancer cell lines. Using RIP and real‐time PCR assays, we observed that immunoprecipitation (IP) of p65 and I*κ*B*α* specifically retrieved NKILA (Fig. 6A and B). Liu et al. demonstrated that NKILA binds to p65 rather than p50 or I*κ*B*α*, and NKILA can only retrieve p50 or I*κ*B*α* from complexes containing p65 in breast cancer cell line 15; herein, we confirmed the combination of NKILA to p65 in laryngeal cancer cell lines.

### NKILA reduces the resistance of the laryngeal cancer cell to X‐ray radiation through inhibiting p65 nuclear translocation

**Figure 7:**
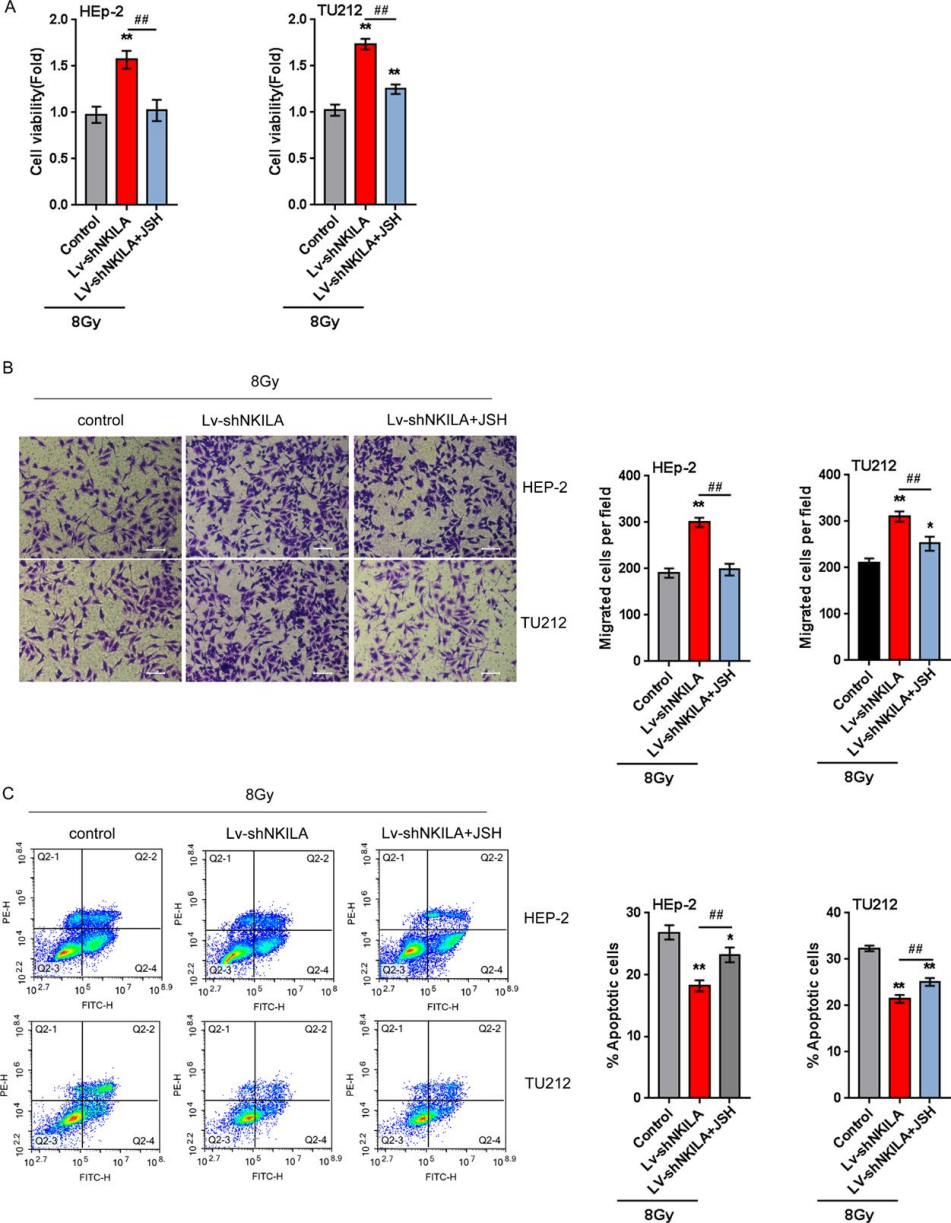
NKILA reduces the resistance of the laryngeal cancer cell to X‐ray radiation through inhibiting p65 nuclear translocation (A) HEp‐2 and TU212 cells were infected with LV‐shNKILA, exposed to 8 Gy for 48 h, and treated with PBS or JSH; the cell viability was determined using MTT assays; (B) the cell migration was determined using Transwell assays. (C) The cell apoptosis was determined using flow cytometer assays. The data are presented as mean ± SD of three independent experiments. *P <* *0.05, **P <* *0.01, ^##^ *P *<0.01.

We have found that NKILA reduces the protein levels of cytoplasm and nucleus p65; next, we validated whether NKILA overexpression could inhibit p65 nuclear translocation, thereby affecting the resistance of the laryngeal cancer cell to X‐ray radiation. HEp‐2 and TU212 cells were infected with LV‐shNKILA and exposed to 8 Gy X‐ray radiation for 48 h in the presence or absence of JSH, an inhibitor of p65 nuclear translocation; the cell viability, migration, and apoptosis were determined using MTT, Transwell, and flow cytometer assays. The results showed that NKILA knockdown promoted cell viability and migration, while reduced cell apoptosis; in the presence of JSH, the effect of NKILA on cell viability, migration, and apoptosis of laryngeal cancer cells were significantly reversed (Fig. 7A–C), indicating that inhibiting p65 nuclear translocation may sensitize laryngeal cancer cell to X‐ray radiation.

### NF‐*κ*B binds to the NKILA promoter region of to activate its transcription

**Figure 8:**
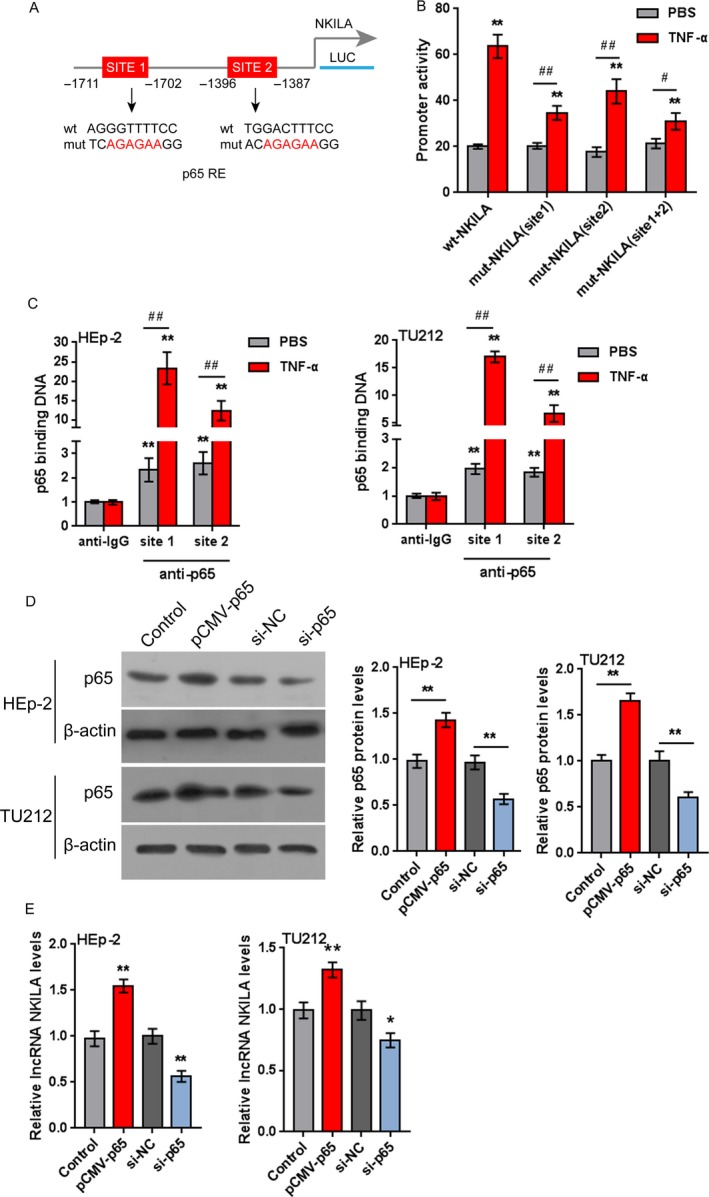
NF‐*κ*B binds to the promoter region of NKILA to activate its transcription (A) A schematic diagram of the potential p65 binding element (two possible binding sites) in the promoter region of NKILA predicted by Jaspar database. A wt‐NKILA promoter luciferase reporter vector and a mut‐NKILA promoter luciferase reporter vector were constructed. (B) The indicated vectors were transfected into HEK293 cells and treated with PBS or TNF‐*α* (10 ng/mL for 24 h); the luciferase activity was determined. (C) The real‐time ChIP assay showed that the level of p65 antibody binding to NKILA promoter was much greater than that of IgG in HEp‐2 and TU212 cells. (D) HEp‐2 and TU212 cells were transfected with pCMV‐p65 or si‐p65 to achieve p65 overexpression or knockdown, as confirmed using Western blot assays. (E) The expression levels of NKILA in the indicated cells were determined using real‐time PCR assays. The data are presented as mean ± SD of three independent experiments. *P <* *0.05, **P <* *0.01 , ^#^ *P* <0.05, ^##^ *P* <0.01.

In order to further validate whether NF‐*κ*B could activate NKILA transcription through binding to its promoter region, luciferase reporter gene assays were employed. Using online tool Jaspar database, we predicted that NKILA promoter possesses two p65‐reactive elements (p65RE, Fig. 8A). Through mutating any of the two putative binding sites, we constructed wild‐type NKILA (named wt‐NKILA, possessing no mutation) and mutant‐type NKILA (named mut‐NKILA, containing a mutation in any or both of the two predicted NF‐*κ*B RE) luciferase reporter gene vectors (Fig. 8A). The indicated vectors were transfected into HEK293 cells and treated with PBS or TNF‐*α*, which has been well‐established to promote NF‐*κ*B expression, and then the luciferase activity was determined. The results showed that TNF‐*α* treatment significantly amplified the luciferase activity of wt‐NKILA as compared to PBS treatment. When any or both of the two putative binding elements were mutated, TNF‐*α*‐induced changes of the luciferase activity were abolished (Fig. 8B). Furthermore, the real‐time ChIP assay showed that the level of p65 antibody binding to either site 1 or site 2 of the binding elements in the NKILA promoter was much greater than that of IgG (Fig. 8C and D), suggesting that p65 might bind to the promoter of NKILA at any of the two predicted sites to activate its expression.

Next, we assessed the effect of p65 overexpression and knockdown on NKILA expression. HEp‐2 and TU212 cells were transfected with pCMV‐p65 or si‐65 to achieve p65 expression, as confirmed using Western blot assays (Fig. 8D); the expression levels of NKILA were then determined using real‐time PCR assays. The results showed that p65 overexpression significantly up regulated NKILA expression while p65 knockdown down regulated NKILA expression in HEp‐2 and TU212 cells (Fig. 8E). The data indicate that NF‐*κ*B binds to the promoter region of NKILA to activate its expression.

**Figure 9:**
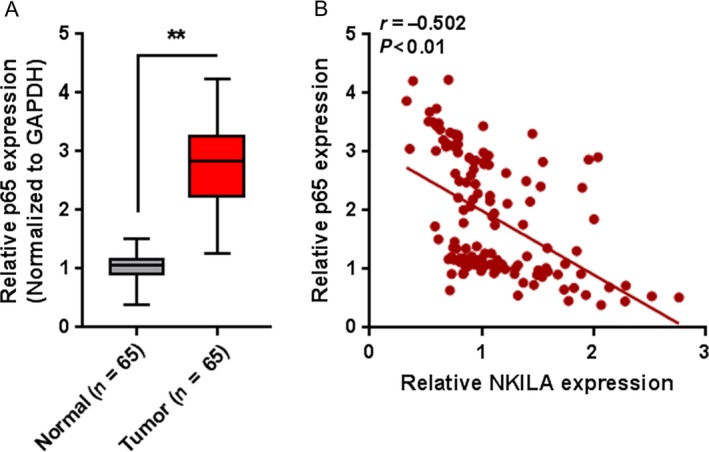
The expression of p65 in tissue samples and its correlation with NKILA (A) The expression levels of p65 in 65 paired tumor and nontumor tissue samples were detected using real‐time PCR assays. The data are presented as mean ± SD of three independent experiments. **P <* *0.01. (B) The correlation between p65 and NKILA was analyzed using Spearman's rank correlation analysis.

To further confirm the above findings, the expression levels of p65 in tumor and nontumor tissue samples were detected using real‐time PCR assays. The results showed that p65 expression was significantly up regulated in tumor tissues compared to that in nontumor tissues (Fig. 9A). Moreover, the expression of p65 and NKILA was negatively correlated (Fig. 9B).

## Discussion

In the present study, we demonstrated that NKILA expression was significantly down regulated in laryngeal cancer tissues, particularly in tissues derived from patients in advanced N stages or clinic stages. The overall survival of patients with low NKILA expression was shorter than that of patients with high NKILA expression. Through combining with NF‐*κ*B: I*κ*B complex, NKILA reduced I*κ*B phosphorylation and NF‐*κ*B activation, inhibited p65 nuclear translocation, thereby sensitizing laryngeal cancer to radiotherapy. Moreover, NF‐*κ*B bound to the promoter region of NKILA to increase NKILA transcriptional activity.

In recent decades, the crucial role of lncRNAs in cancer progression has been extensively studied. lncRNA expression disorders have been found to be associated with many cancers. A large amount of lncRNAs plays a key role in regulating tumor signal pathway, thereby affecting tumorigenesis and progression from many different aspects, including proliferation, invasion, migration, or genomic stability 25. In previous studies, NKILA expression is down regulated in tumor tissues and/or cell lines, including nonsmall cell lung cancer 17 and tongue squamous cell carcinoma 18. Herein, NKILA expression was significantly down regulated in laryngeal cancer; particularly, NKILA expression was much lower in patients with advanced N stages and advanced clinical stages. Patients with low NKILA expression obtained a significantly shorter overall survival, suggesting that the NKILA expression decrease may contribute to the poor prognosis of patient with laryngeal cancer. NKILA overexpression significantly suppressed the cell viability and migration, whereas promoted the apoptosis of laryngeal cancer cell, indicating its potential as a tumor suppressor.

So far, radioresistance of laryngeal cancer still remains one of the huge challenges 1, 2; herein, we assessed the effect of NKILA on the radioresistance of laryngeal cancer to X‐ray radiation. After NKILA knockdown, the cytotoxicity of X‐ray radiation on laryngeal cancer cell was partially reversed, manifested as promoted cell viability and migration and suppressed apoptosis of laryngeal cancer cell. However, the underlying mechanism still needs to be investigated.

lncRNA can serve as a molecular scaffold which interacts with multiple regulatory proteins 26, 27. lncRNA HOTAIR serves as a scaffold for two different histone modification complexes: polycomb repressive complex 2 (PRC2) and LSD1/CoREST/REST complex 27. NRON acts as a molecular scaffolding required for the assembly of NFAT and NFAT kinases, which are responsible for NFAT phosphorylation and chelating in cytoplasm 28. According to previous studies, NKILA combines with NF‐*κ*B: I*κ*B complex and directly mask the phosphorylation site of I*κ*B from IKK, thereby inhibiting IKK‐induced I*κ*B phosphorylation and degradation, finally inhibiting the activation of NF‐*κ*B signaling in breast cancer 15, nonsmall cell lung cancer 17, and tongue squamous cell carcinoma 18. Through inhibiting NF‐*κ*B signaling, NKILA serves as a tumor suppressor in the above three cancers. Herein, we also validated the combination between NKILA and NF‐*κ*B: I*κ*B complex in laryngeal cancer cell lines. Consistent with the above study 15, NKILA significantly reduced p65 protein levels and I*κ*B phosphorylation in laryngeal cancer cell lines. Moreover, NKILA also inhibited p65 nuclear translocation in laryngeal cancer cells exposed to 8 Gy radiation, thereby enhancing the cytotoxicity of X‐ray radiation on cancer cells.

NF‐*κ*B activation has been regarded as a crucial contributor in cancer progression 29, 30. A large amount of negative regulators of NF‐*κ*B signaling can serve as tumor suppressors, including dimethylamino parthenolide (DMAPT) 31, sporamin 32, and several microRNAs 33. Interestingly, not only these regulators affect the activation of NF‐*κ*B signaling, but NF‐*κ*B also can transcribe them in return, resulting in a negative feedback loop that blocks sustained or excessive activation of NF‐*κ*B signaling 34. Herein, we have already confirmed that NKILA negatively regulates NF‐*κ*B signaling; next, we validated whether NF‐*κ*B could also activate NKILA transcription to promote its expression. As expected, the data of luciferase reporter gene and ChIP assays revealed that NF‐*κ*B could bind to the promoter region of NKILA, thereby activating its transcriptional activity and promoting NKILA expression in laryngeal cancer cell lines. Subsequently, upregulated NKILA then inhibits I*κ*B phosphorylation and NF‐*κ*B activation, thus forming a negative feedback loop to sensitize laryngeal cancer cell to X‐ray radiation.

Taken together, we demonstrated that NKILA serves as a tumor suppressor in laryngeal cancer by suppressing laryngeal cancer cell viability and migration, whereas promoting cell apoptosis; NKILA knockdown reverses the cytotoxicity of X‐ray radiation on laryngeal cancer cells through combining with NF‐*κ*B: I*κ*B complex to inhibit I*κ*B phosphorylation, inhibit p65 nuclear translocation, and finally inhibit NF‐*κ*B activation. NF‐*κ*B binds to the promoter region of NKILA to activate its transcriptional activity, upregulated NKILA then inhibits I*κ*B phosphorylation and NF‐*κ*B activation, thus forming a negative feedback loop to sensitize laryngeal cancer cell to X‐ray radiation. NKILA can serve as a promising agent of enhancing the cytotoxicity of X‐ray radiation on laryngeal cancer and addressing the radioresistance of laryngeal cancer.

## Conflict of Interest

None declared.

## Ethics Approval and Consent to Participate

The study was conducted in accordance with the Declaration of Helsinki, and the protocol was approved by the Ethic Committee of The Second Xiangya Hospital, Central South University. All of the enrolled patients signed informed consent forms.

## Supporting information


**Table S1.** The sequences used in the present study.
